# Validation of the Danish version of the disease specific instrument EORTC QLQ-CR38 to assess Health-related quality of life in patients with colorectal cancer

**DOI:** 10.1186/1477-7525-10-150

**Published:** 2012-12-14

**Authors:** Henriette Vind Thaysen, P Jess, Søren Laurberg, M Groenvold

**Affiliations:** 1 Department of Surgery P, Aarhus University Hospital, Tage-Hansens Gade 2, Aarhus C 8000, Denmark; 2 Department of Surgery, Roskilde Hospital, Roskilde, 2400, Denmark; 3 University of Copenhagen, Copenhagen N, 2000, Denmark; 4 Department of Palliative Medicine, Bispebjerg Hospital, Copenhagen NV, 2400, Denmark; 5 Department of Public Health, University of Copenhagen, Copenhagen K, 1014, Denmark

**Keywords:** Health-related quality of life, EORTC QLQ-CR38, Colorectal cancer, Psychometric validation

## Abstract

**Background:**

The European Organisation for Research and Treatment of Cancer (EORTC) quality of life colorectal questionnaire module (QLQ-CR38) was developed in 1999, and an update, the QLQ CR29 was published recently. To date the Danish version of the questionnaire has not been validated. The aim of this study was to examine the psychometric properties of the Danish version of EORTC QLQ-CR38.

**Methods:**

EORTC QLQ-CR38 was administered to 190 patients with colorectal cancer in two Danish hospitals, one month after their operation. A psychometric evaluation of the questionnaire’s structure, reliability, convergent, divergent and known-groups validity was performed.

**Results:**

Data from 164 (86.3%) patients were available for analysis. The Danish version of EORTC QLQ-CR38 showed satisfactory psychometric properties for the scales: body image, sexual functioning, male sexual problems and defecations problems. Suboptimal psychometric performances were found for the scales: micturition problems, symptoms of the gastrointestinal tract and weight loss. Evaluation of the psychometric properties of the scale chemotherapy side effects was limited by the low number of patients receiving chemotherapy. It was not possible to assess the psychometric properties of the scale female sexual problems and the single item sexual enjoyment due to a high number of missing values. The homogeneity of the study population made the evaluation of known-group validity difficult.

**Conclusions:**

The results of this study suggest that the validity of the Danish version of EORTC QLQ-CR38 is acceptable. Furthermore, the results support the appropriateness of the updated version, the EORTC QLQ-CR29.

## Background

Health-related quality of life (HRQoL) has become an important outcome endpoint in cancer research and can be measured by generic and/or disease – specific instruments. A specific instrument for colorectal cancer patients, the European Organisation for Research and Treatment of Cancer (EORTC), quality of life colorectal questionnaire module (QLQ-CR38), was developed in 1999 by EORTC [1]. EORTC QLQ-CR38 was designed as a supplement to the core questionnaire (EORTC QLQ-C30), which assesses HRQoL of patients with cancer, but is not specific for any kind of cancer. EORTC QLQ-CR38 looks into important issues relevant for colorectal cancer, such as bowel, bladder, and sexual dysfunction as well as problems in relation to stoma function. It has been translated into several languages, including Danish. The Danish version was devised based on forward-backward translation and pilot-testing among patients, as described in the guidelines of EORTC [2]. An update and international validation of EORTC QLQ-CR38 were undertaken between 2007–2009, resulting in a shorter version of the questionnaire, EORTC QLQ-CR29 [3,4]. Although the Danish version of EORTC QLQ-CR38 has been used in Danish research, the questionnaire has never been formally validated, which is also the case with other Scandinavian versions.

### Aim

The aim of this study was to examine the psychometric properties of the Danish version of EORTC QLQ-CR38, in post-surgical colorectal cancer settings.

## Methods

### Patients

EORTC QLQ-CR38 and EORTC QLQ-C30 were administrated to patients with colorectal cancer treated at Hillerød Hospital and Aarhus University Hospital approximately one month after their operation. This was carried out as part of their participation in two randomized, international, multi-centre studies: Colorectal cancer laparoscopic or open resection (COLOR II) [5] (only Hillerød Hospital) and COLOFOL [6]. COLOR II was designed to investigate short- and long-term outcomes of open versus laparoscopic surgery for rectal cancer [5]. In COLOFOL the effects of two different frequencies of surveillance programs following radical resection for colorectal cancer on mortality, recurrence-free survival, cost-effectiveness and quality of life were examined [6]. In both studies, the patients completed the questionnaires during a follow-up visit in the outpatient clinic. A research nurse was present to introduce the patients to the questionnaire. The patients were instructed to fill out the questionnaire by themselves. Inclusion criteria were as defined in the two studies [5,6].

Clinical and sociodemographic data were extracted from the Danish Colorectal Cancer Group (DCCG) database. Data for this database are collected by all surgical departments in Denmark and are obtained through questionnaires filled in by the patients and surgeons. Complications were defined as postoperative complications occurring within 30 days after surgery, and were divided into surgical and medical complications. Information concerning the presence of stoma was acquired from the question “yes or no to stoma” from EORTC QLQ-CR38. Data were collected between April 2007 and June 2010. Written consent was obtained when patients first entered COLOR II and COLOFOL.

### Questionnaires

EORTC QLQ-CR38 was developed to be used in conjunction with EORTC QLQ-C30 [7]. It incorporates two functional scales: body image and sexual functioning: and two single items assessing future perspective and sexual enjoyment. The seven symptom scales comprise micturition problems, symptoms in the area of the gastrointestinal tract, chemotherapy side effects, problems with defecation, stoma-related problems, male and female sexual problems, and one single item on weight loss. Completed questionnaires were scored according to the instructions from the EORTC group. Items are scored on a four-point scale from 1(”not at all) to 4 (“very much”). Raw scores are aggregated and converted to a linear scale ranging from 0 to 100, with higher scores representing a higher level of functioning or a higher level of symptoms [8]. Of the 38 items, 19 are applicable for all patients. The remaining 19 items are applicable to sub-groups of patients: males, females, patients who are sexually active, and patients with or without stoma. All questions refer to the previous week, with exception of sexual issues which have a 4-week time frame [1].

### Statistical analysis

Means and medians were both calculated for a more comprehensive description of the data than when only one of these summary statistics is used [9]. Missing answers were dealt with according to the Scoring Manual: if at least half of the items in the scale had been completed, it was assumed that the missing item(s) would have had values equal to the average of the items present [8]. Ceiling and floor effect was explored through proportion of respondents with maximum and minimum scores, respectively.

### Reliability

Internal consistency was evaluated by Cronbach’s alpha coefficient. A coefficient value of at least 0.7 were considered acceptable [10].

### Known-groups comparison

Methods of known-groups comparison were used to assess the ability of the questionnaire to discriminate between subgroups of patients with different demographic and clinical status. Comparison between subgroups was only made where differences were expected [11]. Subgroup comparisons included age (>64 years or ≤ 64 years), gender, type of cancer (colon or rectal cancer), type of surgery (open or laparoscopic), presence of stoma (with or without stoma), The American Society of Anaesthesiologists (ASA) score was assigned by the surgeon and performance status was evaluated by the patient before surgery. Performance status and ASA score were dichotomised. Performance status was divided into “excellent or good” versus “more or less, less good or bad”. ASA scores were divided into normal healthy patients (ASA score I) versus patients with mild to severe systematic disease (ASA score II-III).

Clinical significance is an important issue in the interpretation of differences in HRQoL. For EORTC QLQ-C30 [12,13] Cocks suggested that a range of 3–6 points reflects small differences and 9–19 points indicates medium differences [13]. Such ranges have not been determined for EORTC QLQ-CR38. Based on measurements with a variety of instruments, Fayers suggested that a change of 5–10 points as being perceptible by the patient and deemed by the patients as significant [14]. Accordingly, differences ≥ 5 points were accepted as a clinical significant difference between subgroups in this study. Group differences were assessed using the Mann-Witney U-tests.

### Convergent and discriminant validity

Analysis of convergent and discriminant validity were used to examine whether items fit with their proposed scale. Item convergent validity was defined as an item-scale correlation of 0.40 or greater (corrected for overlap). Item discriminant validity was defined as an item having a higher correlation with its own scale (corrected for overlap) than with another scales [10]. Spearman’s Rho was used.

## Results

### Patients

One hundred and sixty seven (87.9%) of the patients included in COLOR II and COLOFOL answered the EORTC QLQ -CR38 one month after the operation. All questionnaires were filled out by the patient themselves. Clinical and sociodemographic data were missing for three patients. In all, data from 164 patients (86.3%) were available for further analysis.

Clinical and sociodemographic data are shown in Table 1. The median age was 64 years. Seventy-nine (59.2%) had colon cancer and 67 (40.8%) had rectal cancer. Stoma was present in 7.3% patients treated for colon and 68.7% patients treated for rectal cancer.

**Table 1:** Clinical and sociodemographic characteristics of the study sample

**Characteristics n = 164**		**Number (%)**	**CI 95%**
Age (range 39–83)			
-	Mean	63.8	62.4-65.2
-	Median	64.0	
Gender			
-	Female	60 (36.6)	29.1-44.0%
-	Male	104 (63.4)	56.0-70.9%
Civil status			
-	Married	110 (67.1)	
-	Single	28 (17.1)	
-	Widow (er)	26 (15.9)	
Asa-score			
-	I	60 (36.6)	
-	II	90 (54.9)	
-	III	14 (8.5)	
Physical performance			
-	Excellent or good	81 (49.4)	
-	More or less	39 (23.8)	
-	Less good or bad	19 (11.6)	
-	Missing	25 (15.2)	
Cancer type			
-	Colon	97 (59.1)	51.2-66.7%
-	Rectum	67 (40.9)	33.3-48.8%
Surgery			
-	Laparotomy	103 (62.8)	
-	Laparoscopic	61 (37.2)	
Surgery modality			
-	AR	56 (34.2)	
-	APR	14 (8.5)	
-	Right or transverse colon resection	40 (24.4)	
-	Left colon or sigmoideum resection	49 (29.9)	
-	Other	5 (3.1)	
Stoma (temporary and definitive)			
-	Yes	55 (33.5)	24.7-39.4%
-	No	108 (65.9)	56.8–71.9%
-	Missing	1 (0.6)	0,0 -3.4%
Complications			
-	Surgical	25 (15.2)	
-	Medical	9 (5.5)	
T-stadium			
-	T1	6 (3.7)	
-	T2	19 (11.6)	
-	T3	115 (70.1)	
-	T4	23 (14.0)	
-	Missing	1 (0.6)	
Lymph node metastasise			
-	0	90 (54.9)	
-	≥ 1	72 (43.9)	
-	Missing	2 (1.2)	
Postoperative Chemotherapy		50 (30.5)	
-	Missing	14 (8.5)	
Preoperative radiation therapy		12 (7.3)	
-	Missing	24 (14.6)	

* AR: Anterior resection APR: Abdominoperineal extirpation (APR).

It was not possible to obtain data about whether patients had started postoperative chemotherapy when they answered the questionnaire.

### Missing values

The distribution of the answers for each item and scale are shown in Table 2 (first column). The majority of missing data were from items concerning sexuality issues, with 7.9% -11% of the answers to sexual functioning missing. The answers to the sexual enjoyment item as well as the two items concerning male and female sexual problems were conditional on having been sexually active (item 49). Surprisingly, only 39 (37.5%) men answered the item concerning sexual enjoyment, while 82 (78.8%) men answered the questions concerning male sexual problems. There was a much lower response rate to the equivalent questions among women. Only 14 (23.3%) women answered the question about sexual enjoyment, and of those, 11 (18.3%) answered the questions concerning female sexual problems. The questions about female sexual problems could not be further analysed due to the high number of missing values.

**Table 2:** Distribution of scores and Cronbach alpha of EORTC QLQ - CR38 in colorectal cancer patients

**Scale Item number**	**n = 164 (%)**	**Mean (SD)**	**Median**	**Floor %**	**Ceiling %**	**Range**	**Cronbach alpha**
Micturition problems	159 (97.0)	24.3 (19.7)	22.2	22.0	0.6	0-100	0.65
31) Frequency urination day *	158 (96.3)	32.9 (28.4)	33.3	34.8	1.9	0-100	
32) Frequency urination day night *	158 (96.3)	30.2 (27.8)	33	36.1	3.8	0-100	
33) Pain while urinating*	159 (97.0)	10.1 (20.1)	0	76.7	0.6	0-100	
Symptoms gastro-intestinal tract	163 (99.4)	18.9 (15.1)	20	15.3	0	0-80	0.55
34) Bloated feeling in stomach *	161 (98.2)	22.4 (26.8)	0	50.3	3.7	0-100	
35) Abdominal pain *	161 (98.2)	23.0 (24.5)	33.3	46.0	1.2	0-100	
36) Pain in buttocks	162 (98.8)	9.1(22.3)	0	82.7	2.5	0-100	
37) Bothered by gas	162 (98.8)	27.4 (27.5)	33.3	42.0	2.5	0-100	
38) Belching	163 (99.4)	12.9 (22.6	0	71.2	1.2	0-100	
39) Weight loss	162 (98.8)	27.8 (31)	33.3	44.4	8.0	0-100	
Chemotherapy side effects	163 (99.4)	15.8 (16.0)	11.1	33.7	0	0-83.3	0.37
40) Dry mouth*	162 (98.8)	23.7 (28.0)	33.3	64.4	5.0	0-100	
41) Thin or lifeless hair	159 (97.0)	5.7 (15.1)	0	85.5	0.6	0-100	
42) Trouble with taste	163 (99.4)	17.4 (27.6)	0	64.4	5.0	0-100	
Body image *	163 (99.4)	80.4 (22.1)	88.9	1.2	36.8	0-100	0.78
43) Feeling less attractive *	163 (99.4)	80.2 (25.8)	100	2.5	55.8	0-100	
44) Feeling less feminine/masculine *	162 (98.8)	84.4 (26.3)	100	3.7	67.9	0-100	
45) Dissatisfied with body*	163 (99.4)	76.5 (27.7)	66.6	4.2	49.1	0-100	
46) Future perspective *	163 (98.8)	57.5 (27.0)	66.7	6.7	16.0	0-100	
Sexual function	151 (92.1)	20.1 (23.6)	16.7	46.4	0.7	0-100	0.85
47) Interest in sex *	151 (92.1)	23.6 (27.4)	0	50.3	2.0	0-100	
48) Sexual activity	146 (89.0)	16.9 (23.2)	0	60.3	0.7	0-100	
49) Sexual enjoyment	53 (32.3)	54.1 (36.5)	33.3	17.0	30.2	0-100	
Male sexual problems	82 (78.8)	40.2 (38.4)	33.3	29.3	19.5	0-100	0.80
50) Problems erection*	82 (78.8)	45.5 (41.1)	33.3	34.1	24.3	0-100	
51) Problems ejaculation	79 (76.0)	34.2 (42.7)	0	54.4	25.3	0-100	
Female sex problems	11 (18.3)	19.7 (23.4)	16.7	45.5	0	0-66.7	0.35
52) Dry vagina	11 (18.3)	72.7 (36.0)	100	54.5	9.1	0-100	
53) Pain during intercourse	11 (18.3)	12.1 (22.5)	0	72.7	0	0-66.6	
Defecation problems (no stoma)	104 (96.3)	15.9 (13.8)	14.3	12.5	0	0-57.1	0.72
55) Frequent bowel movement day *	104 (96.3)	37.2 (28.8)	33.3	25.0	6.7	0-100	
56) Frequent bowel movement night*	104 (96.3)	17.9 (27.4)	0	63.5	3.9	0-100	
57) Urge without stools	105 (97.2)	22.9 (27.1)	0	50.5	2.9	0-100	
58) Unintentional release of stools	104 (97.2)	8.0 (17.1)	0	79.8	0	0-66.7	
59) Blood with stools *	104 (96.3)	1.3 (6.44)	0	96.2	0	0-33	
60) Difficulty in moving bowels	105 (97.3)	16.2 (23.6)	0	62.9	1.0	0-100	
61) Painful bowel movement	105 (97.3)	8.6 (17.9)	0	79.1	0	0-66.7	
Stoma-related problems	51 (92.7)	25.8 (20.8)	19.0	5.9	0	0-90.5	0.81
62) Afraid stoma noise	51 (92.7)	25.5 (32.4)	0	52.9	7.8	0-100	
63) Afraid smell stools	51 (92.7)	27.5 (35.7)	0	54.9	11.8	0-100	
64) Worry about leakage	51 (92.79	40.5 (33.5)	33.3	27.5	13.7	0-100	
65) Caring for stoma*	50 (90.9)	16.7 (27.1)	0	66.0	3.9	0-100	
66) Irritated skin	51 (92.7)	25.5 (27.1)	33.3	41.2	5.9	0-100	
67) Embarrassment	51 (92.7)	19.6 (27.6)	0	58.8	3.9	0-100	
68) Feeling less complete	52 (94.5)	26.3 (29.8)	33.3	46.2	5.8	0-100	

(SD) Standard deviation.

* Indicates scales and items included in EORTC QLQ-CR29.

### Score distribution

Mean, median, floor and ceiling values of the function and symptom scales are presented in Table 2. The symptom scores were heavily skewed towards the lower end. The maximum score (100) was observed for micturition problems, male sexual problems, and weight loss, but not for other symptom scales. Functional scores were high for body image, while lower values were observed in future perspective, sexual function, and sexual enjoyment. A floor and/or ceiling effect was observed for all subscales and single items.

### Internal consistency

Cronbach’s alpha coefficient for each scale is shown in Table 2. A value exceeding the 0.70 criterion was achieved in the body image, sexual function, male sexual problems, defecation problems, and stoma-related problems scales. The criterion was not met in scales regarding micturition problems (0.65), symptoms of the gastrointestinal tract (0.55), female sexual problems (0.35), or chemotherapy side effects (0.37).

### Convergent and discriminant validity

The results of the convergent and disciminant validity analyses are provided in Table 3. The criterion of 0.40 for convergent validity was fulfilled for all items in three scales: body image, sexual functioning, and male sexual problems. In the remaining scales of micturition problems, symptoms of the gastrointestinal tract, defecation problems, and stoma-related problems, some of the items did not display satisfactory convergent validity. None of the items in the chemotherapy side effects scale met the 0.40 criterion. In terms of discriminant validity, the “irritated skin” item in the stoma-related problems scale had a very low correlation with its own scale but a higher correlation with the body image scale.

**Table 3:** Item convergent and discriminat validity EORTC QLQ-CR38 (Spearman’s RHO)

**Scales Items**	**MI**	**GI**	**CT**	**BI**	**SXS**	**MSX**	**FSX**	**DF**	**STO**
Micturition problems (MI)									
31) Frequency of urination day	**0.56**	0.15	0.07	0.15	0.08	0.31		0.34	0.03
32) Frequency of urination night	**0.56**	0.22	0.16	0.11	0.01	0.16		0.31	0.17
33) Pain while urinating	0.27	0.10	0.04	0.04	0.06	0.22		0.18	0.08
Symptoms gastro-intestinal tract (GI)									
34) Bloated feeling in stomach	0.05	**0.42**	0.18	0.19	0.07	0.07		0.18	0.16
35) Abdominal pain	0.13	0.38	0.24	0.30	0.01	0.01		0.07	0.24
36) Pain in buttocks	0.05	0.06	0.04	0.16	0.04	0.27		0.16	0.20
37) Bothered by gas	0.18	0.31	0.03	0.12	0.10	0.12		0.25	0.27
38) Belching	0.07	0.20	0.22	0.17	0.15	0.32		0.09	0.19
Chemotherapy side effects (CT)									
40) Dry mouth	0.17	0.18	0.29	0.18	0.20	0.25		0.32	0.06
41) Thin or lifeless hair	0.07	0.18	0.14	0.06	0.12	0.08		0.06	0.08
42) Trouble with taste	0.03	0.16	0.29	0.27	0.18	0.14		0.24	0.02
Body image (BI)									
43) Feeling less attractive	0.14	0.28	0.25	**0.59**	0.12	0.32		0.21	**0.43**
44) Feeling less feminine/masculine	0.16	0.21	0.12	**0.66**	0.07	0.38		0.19	0.37
45) Dissatisfied with body	0.14	0.30	0.21	**0.49**	0.03	0.33		0.22	0.29
Sexual function (SXS)									
47) Interest in sex	0.09	0.05	0.26	0.06	**0.71**	0.37		0.09	0.07
48) Sexual activity	0.02	0.05	0.24	0.07	**0.71**	**0.41**		0.00	0.16
Male sexual problems (MSX)									
50) Problems erection	0.23	0.14	0.30	0.39	0.40	**0.66**			
51) Problems ejaculation	0.25	0.10	0.13	0.40	0.34	**0.66**			
Female sexual problems (FSX)									
52) Dry vagina									
53) Pain during intercourse									
Defalcation problems (DF)									
55) Frequent bowel movement day	**0.43**	0.27	0.11	0.12	0.04	0.26		**0.44**	
56) Frequent bowel movement night	0.36	0.18	0.10	0.20	0.02	0.35		**0.45**	
57) Urge without stools	0.21	0.21	0.26	0.26	0.12	0.38		**0.56**	
58) Unintentional release of stools	0.21	0.22	0.20	0.14	0.14	0.34		0.36	
59) Blood with stools	0.23	0.17	0.10	0.08	0.02	0.02		0.22	
60) Difficulty in moving bowels	0.02	0.10	0.25	0.15	0.01	0.24		0.27	
61) Painful bowel movement	0.21	0.14	0.26	0.11	0.04	0.13		0.30	
Stoma-related problems (STO)									
62) Afraid stoma noise	0.28	**0.44**	0.16	**0.43**	0.20				**0.56**
63) Afraid smell stools	0.35	0.36	0.06	**0.48**	0.04				**0.60**
64) Worry about leakage	0.18	0.18	0.20	0.33	0.11				**0.59**
65) Caring for stoma	0.12	0.03	0.18	0.08	0.09				0.35
66) Irritated skin	0.07	0.08	0.12	**0.41**	0.26				0.06
67) Embarrassment	0.09	0.29	0.13	**0.48**	0.12				**0.57**
68) Feeling less complete	0.13	0.28	0.10	**0.43**	0.02				**0.60**

Bolding indicate correlation coefficient > 0.40.

Thickly outlined boxes indicate correlation with own scale corrected for over-lap.

Analysis were not possible for the scales female sexual (FSX) problems n < 11 and male sexual problems (MSX) correlated with stoma-related problems (STO) n ≤ 28.

### Known-groups comparison

Thirteen (50.0%) out of 26 comparisons distinguished between clinical and demographic variables subgroups of patients, with differences of five points or more. Mean scores of the sub-groups are shown in Table 4. For three of the scales, namely body image, male sexual problems and defecation problems, the majority of comparisons distinguished between the defined subgroups as anticipated. However, unexpectedly, a higher degree of defecation problems was found in patients aged ≤ 64 than in the older group. Anticipated differences in sexual functioning were only found in three out of five sub-group comparisons. A lower score was expected for patients with a stoma and patient treated with open surgery, but this was not found. The symptoms of the gastrointestinal tract scale could only distinguish between subgroups in one of two comparisons. As expected, patients treated with open surgery had a higher score compared with patients treated with laparoscopic surgery. A lower score for patients with rectal cancer was expected but not found. For micturition problems and future perspective scales, only one out of three anticipated differences were found. Difference in future perspective was only found amongst patients with different physical performance levels, where patients with better performance status also had better future perspective. The micturition problem scale could only distinguish between genders. Anticipated differences in relation to weight loss were not found in any comparisons.

**Table 4:** Known-groups comparison for the relevant scales

**Scales (Item number)**	**Age**		**Gender**		**Physical performance**		**Type cancer**		**Stoma**		**Type surgery**		**Physical status**	
	**<64 years vs. >64 years**	**P- value**	**Male vs. female**	**P-value**	**Excellent or good vs. less good or bad**	**P-value**	**Colon vs. rectum**	**P-value**	**No stoma vs. stoma**	**P-value**	**Open vs. laparoskopic**	**P-value**	**ASA 1 vs. ASA 2-3**	**P-value**
	**n = 77/87**		**n = 104/60**		**n = 81/57**		**n = 87/21**		**n = 110/53**		**n = 101/61**		**n = 55/99**	
Micturition problems (31–33)	26 vs. 23	0.17	**28 vs. 18**	**0.001**			23 vs. 24	0.96						
Symptoms gastro- intestinal tract (34–38)							20 vs. 18	0.14			**22 vs. 14**	**0.007**		
Weight loss (39)	13 vs. 21	0.07					26 vs. 19	0.46			29 vs. 26	0.9		
Chemotherapy side effects (40–42)														
Body image (43–45)					**84 vs. 75**	**0.02**			**84 vs. 73**	**0.001**	78 vs. 83	0.07		
Future Perspective (46)	55 vs. 60	0.45			**65 vs. 50**	**0.001**							57 vs. 58	0.88
Sexual functioning (47–48)	**29 vs. 12**	**0.001**	**25 vs. 12**	**0.000**					22 vs. 17	0.07	19 vs. 24	0.12	**16 vs. 29**	**0.001**
Sexual enjoyment (49)														
Male sexual problems (50–51)	**28 vs. 52**	**0.008**							**27 vs. 63**	**0.001**	**50 vs. 23**	**0.002**	29 vs. 44	0.12
Female sexual problems (52–53)														
Defecation problems (55–61)	**20 vs. 12**	**0.009**	18 vs. 13	0.2			**14 vs. 24**	**0.004**						
Stoma-related problems (62–68)														

Mean score and P-value for between-groups differences.

Bolding indicates between-groups differences ≥ 5 points and a P-value < 0.05 (Mann-Witney U-tests).

## Discussion

This study examines the psychometric properties of EORTC QLQ-CR38 in a sample of 164 Danish patients with colorectal cancer, who were assessed one month after primary open or laparoscopic surgery. In this study data were obtained from two clinical studies one month after operation. The sample included patients with or without stoma and patients treated with different types of surgical procedures. The proportion of patients treated with laparoscopic surgery was high in this study compared with any other validation study of EORTC QLQ-CR38 [15-17]. A postoperative setting was chosen, in order to examine the performance of the questionnaire in reflecting function and symptoms after surgical treatment. Known-groups comparisons were conducted based on carefully prespecified hypotheses.

The body image, sexual functioning, defecation problems and male sexual problems scales could discriminate between the majority of the defined subgroups. These scales also had a high convergent validity except for the scale concerning defecation problems, which was similar to the original validation. In the original study, convergent, and discriminant analysis confirmed the structure of the functional scales, but did not strongly support the structure of the symptoms scales [1]. With regards to internal consistency, three of the symptoms scales, namely micturition problems, symptoms of the gastrointestinal tract, and chemotherapy side effects had low Cronbach`s alpha coefficients (0.37-0.65), but this is comparable to the original validation [1] and some other subsequent studies [16,17]. In previous studies, the psychometric problems shown in the symptoms scales have been attributed to the fact that symptoms and side-effects do not necessarily occur together, which makes it difficult to cluster items in a meaningful way [17], and thus raising doubt about the usefulness of these scales.

A limitation of this study could be the homogeneity of the population in relation to disease stage, disease severity and performance status. This may underlie a narrower response range and lead to low data variability, which could have compromised the known-group comparison results. It is well-known that the strength of correlations, including Cronbach’s alpha, increases with data variability. The low variability in this data set, reflected in the skewness towards high functional scores and low symptoms scores, may partially account for the less than ideal validity results. A low correlation between items and scales was particularly evident in some symptom scales: symptoms of the gastrointestinal tract, the pain while urinating item in the micturition problems scale and all items concerning chemotherapy side effects. The low correlation for chemotherapy side effects was probably attributable to the low proportion of patients undergoing chemotherapy.

The weight loss item could not distinguish between any of the subgroups. A floor effect was observed for this item, indicating low variability. This could be due to the time reference in the item. The patient is asked to answer the question in relation to the past week, whereas weight loss may have occurred prior to that. This item also demonstrated a low validity in another validation study and has been eliminated from the updated version [16].

It was not possible to carry out known-groups comparison for stoma related problems. The stoma related problems scale is only applicable to patients with stoma, and the number of such patients was too small for further division into subgroups.

The amount of missing data associated with the sexual problems and enjoyment scales rendered known-groups comparison analysis impossible. These questions are conditional on the patient being sexually active. The original EORTC QLQ-CR38 validation study also found a high rate of missing values in these questions, where both male (3%) and female (12%) found them too intrusive and chose not to answer [1]. Since there is no specific question asking whether the patient is sexually active, reasons for non-response could not be differentiated (sexually inactive versus unwillingness to answer). In addition, at just one month after surgery, the proportion of sexually active patients may have been lower than usual. Reluctance of women to respond to questions about their sex life has been well described, which may partially account for the especially high missing values seen in the female sexual problems scale [15,16,18,19].

The capability of the Danish version of EORTC QLQ-CR38 to capture changes over time was not examined in this study, but responsiveness is an important feature of a questionnaire. Responsiveness has been examined in some of the studies validating EORTC QLQ-CR38 [1,15]. The body image, defecation problems, micturition problems, and symptoms of the gastrointestinal tract scales have been reported responsive to treatment-induced change over time. Lack of responsiveness has been reported for the chemotherapy side-effects scale [1,15,20]. One study found the EORTC QLQ-C30 to be more responsive in patients receiving chemotherapy than the EORTC QLQ-CR38 [20]. In addition to the validation studies, some studies have used EORTC QLQ-CR38 for prospective follow-up. The same scales as in the validation studies as well as the scales addressing male sexual problems, future perspective, weight loss, and chemotherapy side-effects were found to be able to detect relevant differences over time in different treatment groups of patients with colorectal cancer [21-29]. Thus, considerable evidence for the responsiveness to change has been found.

There were several reasons for the revision of EORTC QLQ-CR38 into the shorter version EORTC QLQ-CR29. These included changes in the treatment of colorectal cancer, problems with missing data, and that the EORTC QLQ-CR38 contains scales that are unique to subgroups of patients [3,4]. The items and scales from EORTC QLQ-CR38 which remained unchanged in the EORTC QLQ-CR29 are highlighted in Table 2. The EORTC QLQ-CR29 contains 17 unchanged items from EORTC QLQ-CR38, five reworded items, and seven new items [3,4]. The EORTC QLQ-CR29 can be summarized into four scales: body image, urinary frequency, blood or mucus and stool frequency. The body image scale was the only scale that remained unchanged from the EORTC QLQ-CR38, a decision that is supported by its satisfactory psychometric performance found in this study. The micturition problems scale was changed to urinary frequency, and the pain with urination item was removed from the scale due to poor convergent validity, a finding which was replicated in this study. The symptoms in gastro-intestinal tract scale was split into single items (some items were removed and some were reworded). The results of this study support the alteration of both micturition problems scale and the gastro-intestinal tract scale, due to their low convergent validity and known-group validity. The scales concerning stoma and defecation problems were split into single items and were standardised, so that patient response could be compared where possible. The question concerning weight loss has been re-worded from:” Have you lost weight?” to “Have you worried about your weight?” Again, this is supported by this study, as the change may make it easier for the patient to answer. The psychometric properties of the scales concerning sexual functioning and male sexual problems were good, but the amounts of missing values were high. In the EORTC QLQ-CR29, several changes have been made with the aim of reducing missing data in the sexual domain. First, items in this domain have been cut down to two items for men and two items for women: one of which addresses interest in sex and the other addresses sexual problems. Second, the answers are not conditional on being sexually active. Third, these items are moved to the end of questionnaire. These changes may reduce missing values, but at the same time would also decrease the information related to sexual functioning after treatment for colorectal cancer, which is an important aspect of outcome.

## Conclusions

In conclusion, the Danish version of EORTC QLQ-CR38 showed satisfactory psychometric properties for the scales concerning body image, sexual functioning, male sexual problems and defecations problems. Reduced psychometric properties were found especially for the following symptom scales: micturition problems, symptoms of the gastrointestinal tract, and weight loss, which could partially be explained by the homogeneity of the study population. Although the stoma related problems scale showed good internal consistency and convergent validity, no clear conclusion could be drawn regarding its psychometric performance, as known-groups comparison could not be conducted. Evaluation of the psychometric properties of the scale chemotherapy side effects was limited by the low number for patients receiving chemotherapy. It was not possible to assess the psychometric properties of the female sex problems and sexual enjoyment scales due to a high number of missing values.

According to the results of this study, the update of EORTC QLQ-CR38 to the EORTC QLQ-CR29 seems appropriate [3,4]. The results also suggest that EORTC QLQ-CR29 is likely to be a more valid instrument. Nevertheless, a proper validation of this questionnaire in a Danish setting is required.

## Abbreviations

HRQoL: Health-related quality of life; EORTC: European organisation for research and treatment; EORTC QLQ-C30: Quality of life core questionnaire; EORTC QLQ-CR38: Quality of life colorectal questionnaire; EORTC QLQ-CR29: Quality of life colorectal questionnaire; COLOR II: Colorectal cancer laparoscopic or open resection; DCCG: Danish colorectal cancer group; ASA score: The American society of anaesthesiologists score.

## Competing interests

The authors declare that they have no competing interests.

## Authors' contributions

All four authors were involved in drafting the article or critically revising it for important intellectual content, and all authors approved the final version to be submitted for publication. PJ, MG and HVT designed the study. Data collection was made by PJ, SL and HVT. Analysis and interpretation of the results PJ, MG and HVT. The statistical analysis was managed by HVT.
